# M cell targeting by a Claudin 4 targeting peptide can enhance mucosal IgA responses

**DOI:** 10.1186/1472-6750-12-7

**Published:** 2012-03-13

**Authors:** David D Lo, Jun Ling, A Holly Eckelhoefer

**Affiliations:** 1 Division of Biomedical Sciences, University of California, Riverside CA 92521, USA; 2 Department of Basic Sciences, The Commonwealth Medical College, Scranton PA 18510, USA

## Abstract

**Background:**

Mucosal immune surveillance is thought to be largely achieved through uptake by specialized epithelial M cells. We recently identified Claudin 4 as an M cell target receptor and developed a Claudin 4 targeting peptide (CPE) that can mediate uptake of nanoparticles through Nasal Associated Lymphoid Tissue (NALT) M cells.

**Methods:**

Recombinant influenza hemagglutinin (HA) and a version with the CPE peptide at the C-terminal end was used to immunize mice by the intranasal route along with a single dose of cholera toxin as an adjuvant. Serum and mucosal IgG and IgA responses were tested for reactivity to HA.

**Results:**

We found that the recombinant HA was immunogenic on intranasal administration, and inclusion of the CPE targeting peptide induced higher mucosal IgA responses. This mucosal administration also induced systemic serum IgG responses with Th2 skewing, but targeting did not enhance IgG responses, suggesting that the IgG response to mucosal immunization is independent of the effects of CPE M cell targeting.

**Conclusions:**

M cell targeting mediated by a Claudin 4-specific targeting peptide can enhance mucosal IgA responses above the response to non-targeted mucosal antigen. Since Claudin 4 has also been found to be regulated in human Peyer's patch M cells, the CPE targeting peptide could be a reasonable platform delivery technology for mucosal vaccination.

## Background

Most infectious agents enter the body through mucosal surfaces such as the intestine or airways. Protective immune responses induced by such infections involve both cellular immune responses and systemic IgG, but at mucosal surfaces secretory IgA provides the most effective protection. Studies have indicated that IgA responses are dependent on immune responses in mucosal lymphoid tissues such as intestinal Peyer's patches and Nasal Associated Lymphoid Tissues (NALT) or tonsils [1-4], where epithelial M cells acquire and transport antigens to underlying lymphoid tissue. Unfortunately, conventional vaccines rely instead on injected antigens, which induce IgG but not IgA. Live attenuated virus vaccines such as cold-adapted influenza (e.g., FluMist^®^), or oral polio vaccine can provide better mucosal immunity, but these are a greater challenge to develop, and they require an expensive cold chain that complicates delivery in developing countries.

Vaccination at mucosal surfaces is a strategy that can help overcome the limitations of injected vaccines (needle disposal, trained medical staff required to administer the vaccine), but also to provide the benefit of mucosal IgA responses. Progress with this strategy has been made in animal studies using two distinct approaches that could be described as bioengineering versus immunological. In typical bioengineering approaches, vaccine antigens are encapsulated in polymer nanoparticles to package and protect the antigen (reviewed in [5]); the particles are administered in an aerosol suspension for inhalation, or as a liquid suspension for intranasal instillation. Here, it is assumed that M cells will non-specifically acquire the encapsulated antigens from the lumen and initiate mucosal immune responses. However, antigen can also be acquired by dendritic cells in the mucosal epithelium [6,7] and drain into other lymphoid tissues, so mucosal IgA responses are not always efficiently induced.

In contrast to bioengineering strategies, immunological approaches are based on targeting antigen delivery to M cells for specific uptake; direct targeting should provide greater control over the induced immune response than unregulated transport to draining lymph nodes. In animal models, targeting to M cells has been successful in inducing mucosal IgA responses. M cell targeting was achieved using a variety of ligands, including lectins or antibodies specific to a fucose moiety presented at the surface of mouse (but not human) M cells [8-10], RGD peptides to bind exposed integrins [11], and a Reovirus sigma protein specific for JAM-A [12-14]. Challenges still remain, such as the identification of M cell target receptors that will reliably work in humans, and the identification of an effective mucosal adjuvant. Indeed, in the absence of an effective adjuvant, M cell targeting in mice has been found to be very effective in inducing immunological tolerance instead of immunity [12,13].

We previously identified the tight junction protein Claudin 4 as a candidate M cell endocytosis receptor [15-17]. Though Claudin 4 is normally found in tight junctions, it was also found redistributed into the cytoplasm of mouse and human M cells and appears to be part of the particle endocytosis machinery. To test the potential of Claudin 4 targeting, we developed a peptide derived from the c-terminal domain of the *Clostridium perfringens *enterotoxin (CPE), which binds to the second external domain of Claudin 4 [18,19]. Using fluorescently labeled microparticles and polymer nanoparticles displaying CPE or fusion proteins with CPE, we demonstrated that the CPE peptide retains Claudin 4 binding [20] and mediates enhanced uptake by M cells in vivo [21,22]. In addition, CD137 mutant mice that lack M cell function failed to take up Claudin 4-targeted particles, confirming the M cell-dependent uptake [23]. Thus, using the CPE peptide, M cell targeting of mucosal vaccines might be possible in humans.

In the present study, we tested the mucosal immune response to engineered vaccine fusion proteins incorporating antigen and the CPE M cell targeting peptide. We report here that with an intranasal administration protocol, M cell targeted fusion proteins are effective in enhancing secretory IgA responses along with a systemic serum Th2-skewed IgG response.

## Methods

### Recombinant antigens

Soluble HA [24] and fusions with fibritin [25], a c-terminal flagellin and/or CPE30 were designed as His-tagged proteins, and were produced using a Baculovirus expression kit (Invitrogen) in which cloned DNA was transfected into insect SF21 or SF9 cells grown in HyClone SFX-Insect media (Thermo). The insect cells that secrete the protein were then grown at controlled 27°C and the supernatant harvested after incubation. The insect culture media was filtered before purification. Western blot (Invitrogen) was used to verify protein expression and quality before precipitating the media with 80% saturated ammonium sulfate (Fisher). After 2 hours of precipitation at 4°C, protein was centrifuged at 13,000 rpm for 30 minutes to obtain a pellet for processing. The resuspended protein was dialyzed overnight in a PBS 1x solution before binding to HisPur resin (Thermo Scientific) for 2 hours. After washing, the protein was then eluted from resin using increased concentrations of imidazole. Resulting elutant was then dialyzed again in a solution of 0.1X PBS/9 mM HEPES pH 7.4 containing 0.05% Tween-20 (Fisher) before concentrating down to a desired level. Final western blots and Coomassie (Pierce) stained gels were run on each protein to insure quality while concentration was measured using a spectrophotometer and BSA standards.

For studies on protein-peptide conjugates, CPE30 peptide was conjugated to HA antigen using a CPE30 peptide synthesized with a c-terminal GGGGSGGGGS linker. This peptide was then chemically linked to HA at a 10:1 peptide:HA protein ratio, using EDC/Sulfo-NHS (Pierce) activated peptides to link to available amine groups on the HA protein.

### Immunization

BALB/c mice were maintained under Specific Pathogen Free colony conditions at the UC Riverside vivarium. All procedures were performed in accordance with institutional and NIH guidelines. Immunization was performed by instillation of vaccine protein solutions intranasally into anesthetized mice. A volume of 10 microliters was instilled into each nostril (total 20 microliters per mouse). Serum titers were assayed from peripheral blood collected by retro-orbital puncture at the time points indicated. For fecal antibody analysis, dry fecal pellets were weighed, and extracted in a proportionate amount of extraction buffer (1 ml PBS with 0.1 mg/ml trypsin inhibitor per 100 mg fecal pellet). After incubation and centrifugation, 400 microliters of supernatant was mixed with 100 microliters glycerol/1 mM PMSF for storage. Broncho-Alveolar Lavage was taken by flushing lungs with 1.0 ml PBS. In all experimental groups, five mice were used per group. Mice were humanely killed under anesthesia at the conclusion of the experiment.

### Elisa

Black flat-bottom plates (Costar) were coated with recombinant HA or synthetic CPE peptide prepared as described at 10 ug/ml in Coating Buffer (25 mM Na_2_CO_3_, 75 mM NaHCO_3_, pH 9.5). Plates were washed 3x in 1x TBST (50 mM Tris pH 7.5, 0.28 M NaCl, 6 mM KCl, 0.1% Tween 20). using a Biotek ELx405 Automated Plate Washer. Plates were blocked with 3% normal goat serum in 1X PBS, then washed as before. Samples were diluted in blocking buffer, (1:2000 for serum or 1:10 for Feces and BAL), then serially two-fold, and added to the coated and blocked plate in triplicate. After washing, detection was performed with either Rat anti-mouse IgA-AP (Southern Biotech), Goat anti-mouse IgG-AP (Southern Biotech), diluted 1:1000 and 1:2000, respectively, in 1X TBST. For IgG isotype analysis, detection was performed using Goat anti-mouse IgG1-AP (Southern Biotech) or Goat anti-mouse IgG2a-AP (Southern Biotech), diluted 1:2000, in 1X TBST. For final development, 10 mM 4-MUP (Molecular Probes) in DMSO diluted 1:25 in substrate buffer (50 mM K_2_CO_3 _2 mM MgCl_2_, pH 9.8) was added. Fluorescence was detected 90 minutes later at ex 360/em 460 on Molecular Devices SpectraMax M2e plate reader.

### Statistical analysis

Except when noted, ELISA fluorescence values were shown after background signal (equivalent dilution of preimmune sample) was subtracted. As noted in the text, analysis of the ELISA titers was performed by taking fluorescence values in the linear range of the titration curve. Statistical comparisons (five mice per group in all studies) were performed using a one-tailed Mann-Whitney test (Prism, GraphPad Software), on the rationale that a non-parametric test would best measure the consistent effect of the specific vaccine formulations (i.e., addition of the CPE targeting peptide), though similar results were obtained using a *t*-test.

## Results

### Vaccination with CPE conjugated to HA

**Figure 1:**
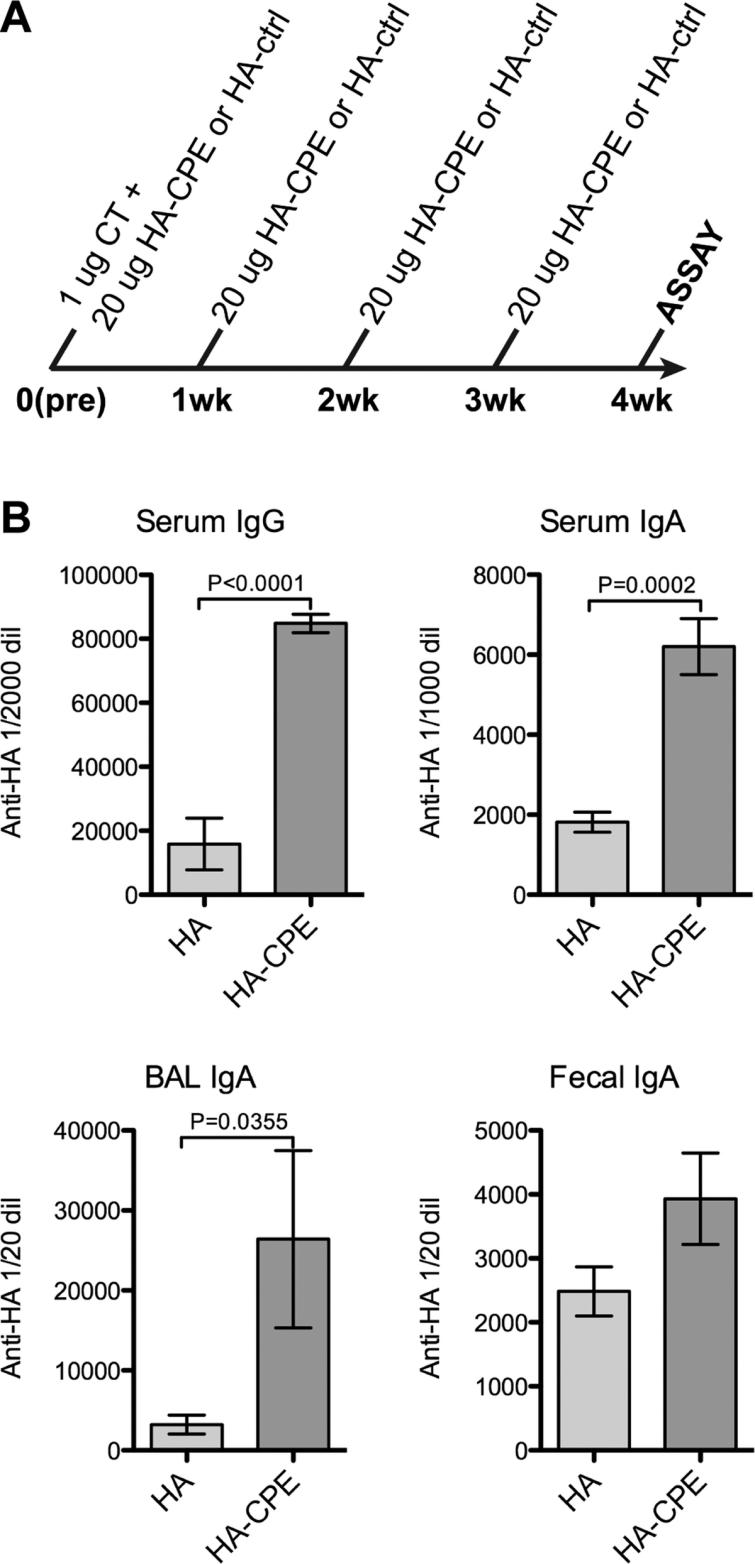
**Immunization with chemical conjugation of CPE to HA antigen**. Basic protocol using HA antigen chemically conjugated to the CPE targeting peptide, showing induction of both systemic serum and mucosal antibody responses. A, Immunization time line. B, ELISA results, showing fluorescence units of anti-HA response at specified dilutions.

To test the ability of CPE-mediated M cell targeting to induce mucosal immunity, we chemically conjugated the Claudin 4-targeting CPE peptide to recombinant influenza hemagglutinin (HA; extracellular domain only, truncated at the transmembrane domain - aa 1-528 [24]), and delivered this antigen intranasally along with cholera toxin in the first dose as an adjuvant. The conjugation of the peptide was accomplished by synthesizing the CPE peptide with a c-terminal linker peptide, and then conjugating this to the HA protein with amide linkages to available lysines. At the conjugation ratio used in this study, there were approximately ten CPE peptides conjugated per trimeric HA complex (or ~3 CPE peptides per monomer). We used a three dose protocol with 1 microgram cholera holotoxin in the first dose as an adjuvant, and 20 micrograms antigen per dose (Figure 1A). The response to a lower dose (2 microgram per dose) was very low (not shown), while the response to the high dose was robust, so it is likely that the chemical conjugation had detrimental effects on the antigenicity of the recombinant HA protein.

Figure 1B shows ELISA results for serum, lung Broncho-Alveolar Lavage (BAL), and extracts from fecal pellets IgA responses to HA. The full titration curves showed that the serum IgA response to HA conjugated with CPE was consistently higher than to the HA alone. However, since endpoint titer calculations could be variable, we chose a point in the titration showing a relatively linear titration in response, and used those values to perform direct comparisons among groups for statistical tests.

### HA-CPE fusion protein vaccine

**Figure 2:**
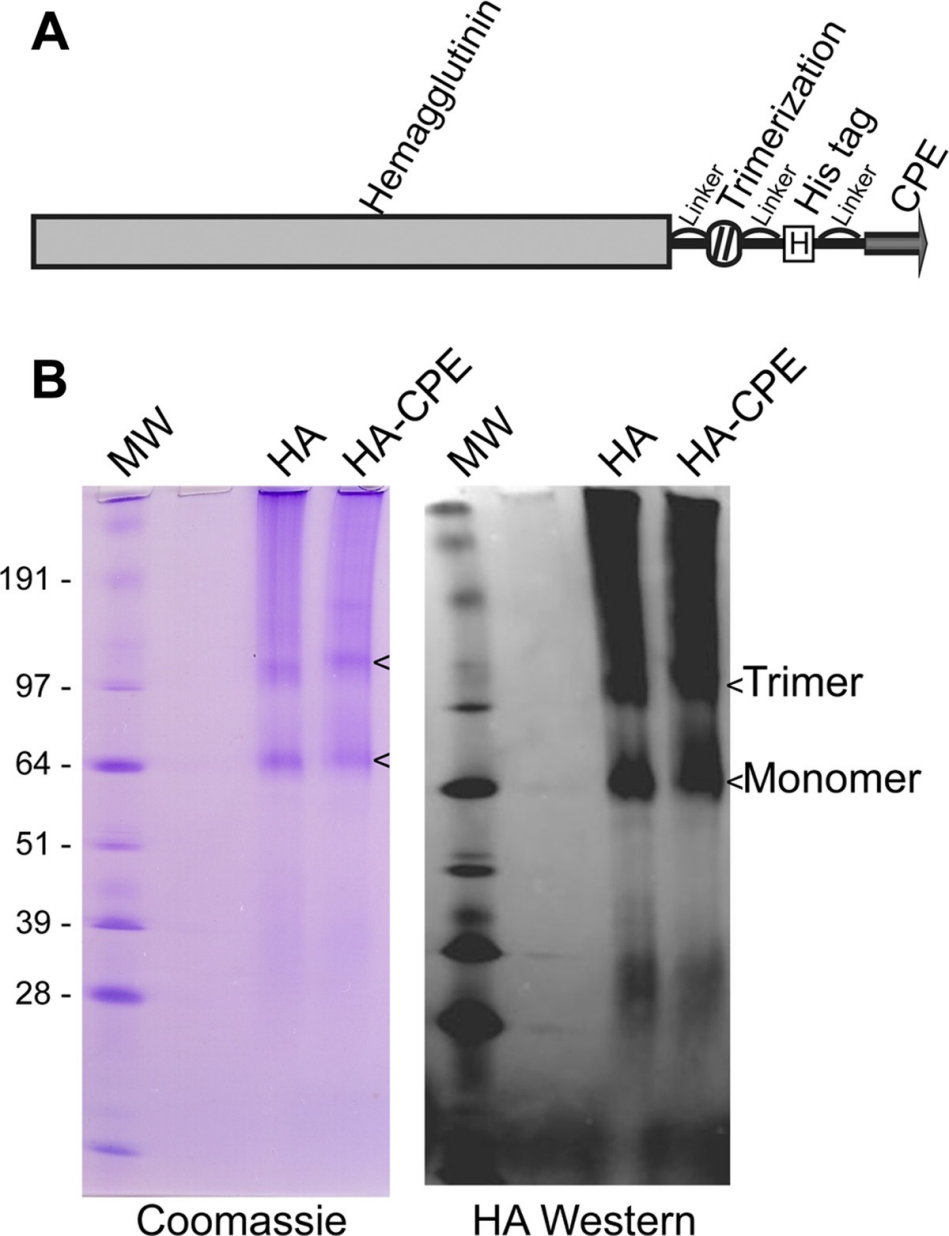
**Recombinant HA trimeric fusion protein**. Development of a hybrid recombinant HA fusion protein vaccine incorporating the CPE targeting peptide. The recombinant HA protein forms trimeric complexes detectable in non-denaturing gels. A, Schematic of the recombinant fusion, as detailed in the text. B, Non-denaturing gels with Coomassie stain and Western blot for HA, showing trimeric and monomeric forms of the protein. Note that in non-denaturing gels, the protein molecular weight markers are not as accurate as in denaturing gels, so the molecular weight comparisons are only relative.

To simplify the production of the vaccine antigen, we developed expression constructs in which the CPE peptide is linked at the c-terminal end of a recombinant fusion protein. Since the influenza hemagglutinin is normally present on the virus particle as a trimer, we included a trimerization peptide from fibritin [25] to stabilize the HA trimer. To separate the functional domains, a peptide linker sequence (GGGGSGGGGSGGGGS) was included. The final protein (Figure 2A) therefore had the structure: [HA] - linker - [trimerization peptide] - linker - [His tag] - linker - [CPE]. For simplicity, this protein will be referred to as HA-CPE, while the control lacking the CPE peptide domain will be referred to as HA. Figure 2B shows non-denaturing Coomassie gel of the purified protein, and blots showing that the purified protein had intact His tag (not shown) and HA determinants, both detectable by Western blot. In these non-denaturing gels the molecular weight markers are not as reliable, but the trimeric complex was still clearly evident as a band at a higher molecular weight. The control HA antigen used was the same except for the absence of the c-terminal linker and CPE domain.

**Figure 3:**
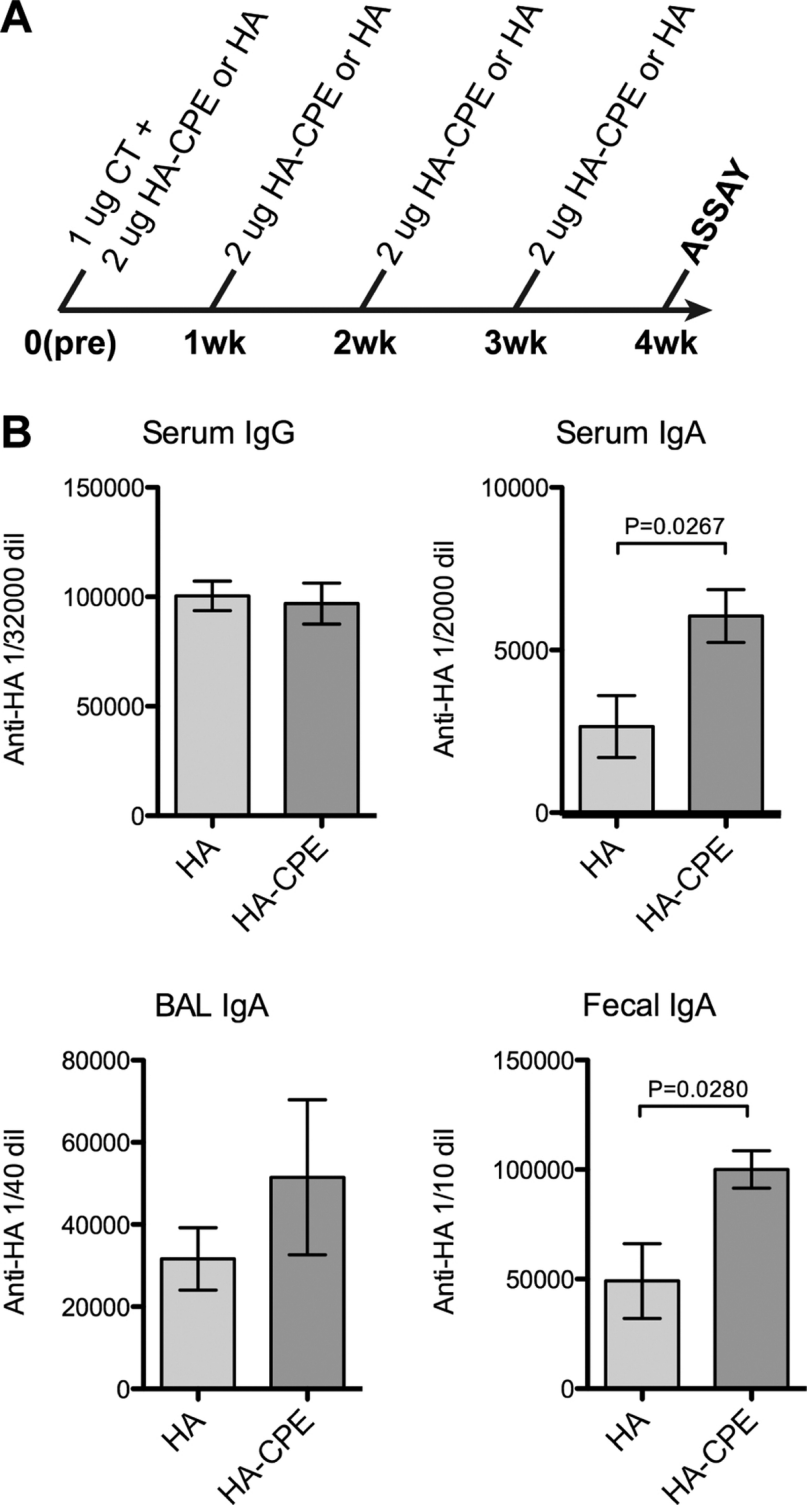
**Immune response to Recombinant fusion protein**. Recombinant protein vaccine and a basic four week intranasal immunization protocol, showing enhanced IgA responses by CPE targeting. Systemic serum IgG responses were also induced, without improvement by CPE targeting. A, Immunization protocol. B, ELISA results, showing increased IgA response to HA-CPE protein.

In this experiment (Figure 3A), a four dose protocol was used; 2 micrograms of the recombinant antigen was given intranasally, with 1 microgram cholera holotoxin in the first dose. Three boosters with antigen alone followed, given in weekly intervals. One week after the last dose, samples were collected from serum lung lavage, and fecal pellets. In this experiment (Figure 3B; shown with background subtracted), the two groups of animals produced similar levels of IgG anti-HA responses in serum, but the targeted HA-CPE antigen induced a significantly higher IgA response in both serum and fecal pellets. The BAL showed similar responses to both antigens though the mean response to targeted vaccine was higher. Thus, with similar recombinant protein antigens, the presence of the M cell targeting peptide CPE induced stronger IgA responses in both serum and the mucosal tissues. Despite the enhanced IgA response to HA, we were unable to detect any antibody response to the CPE peptide, when tested against synthetic CPE peptide bound directly to ELISA plates (not shown).

### Persistence of mucosal IgA response

**Figure 4:**
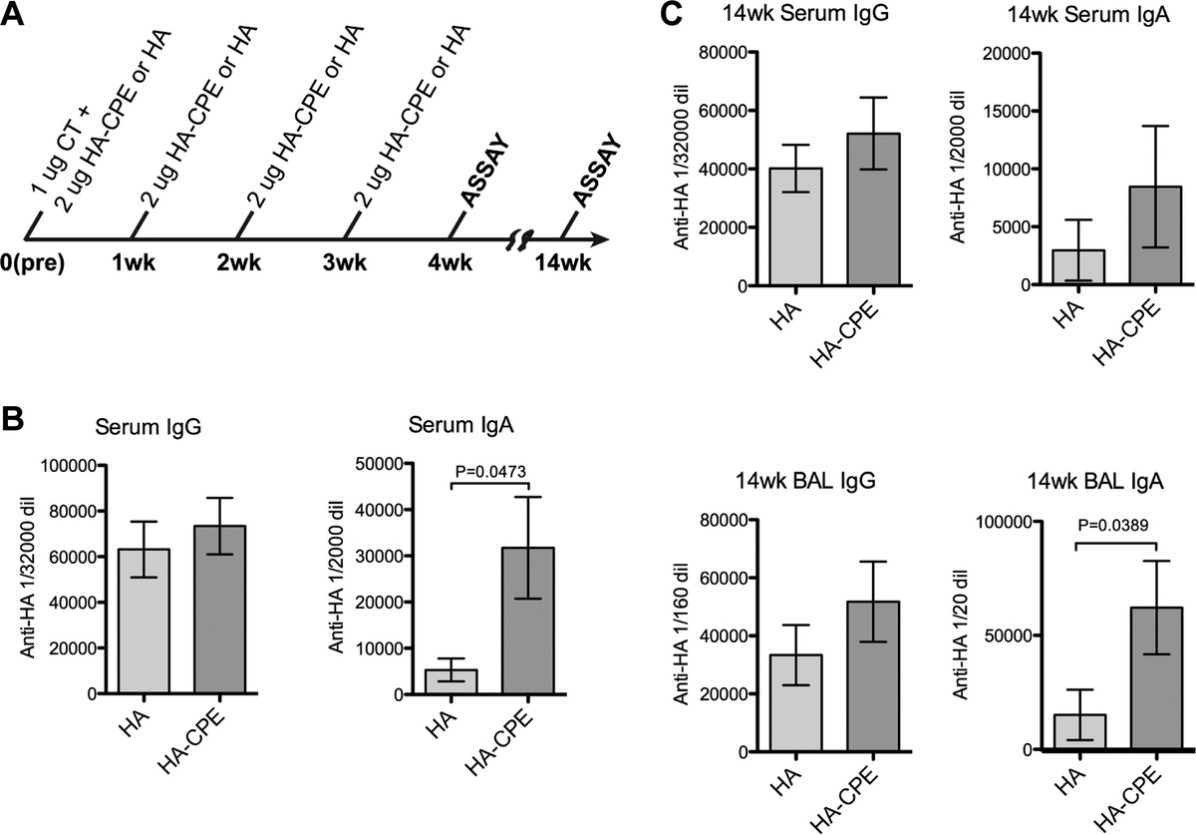
**Persistence of response to recombinant protein vaccine, four dose protocol**. After an initial four week course of immunization, mice were rested for ten weeks for later assay. Results showed persistence of both the antibody response and evidence for enhanced IgA response when CPE targeting was used. A, Immunization protocol. B, Response 4 weeks after first dose. C, Response 14 weeks after first dose.

To assess the persistence of mucosal IgA responses using this vaccine in a four week protocol, mice were tested at 4 weeks after the first dose (Figure 4A) then tested a second time, 14 weeks after the initial dose (Figure 4A). In both groups, significant responses to HA were detected at the 14 week time point. The increased serum IgA response to targeted antigen seen at 4 weeks after the first dose (Figure 4B) was less evident at 14 weeks (Figure 4C), but an increased BAL IgA response was present at the 14 week time point. Thus, a persistent mucosal response to intranasal immunization was detected after 14 weeks, with some enhancement induced by the targeted vaccination.

**Figure 5:**
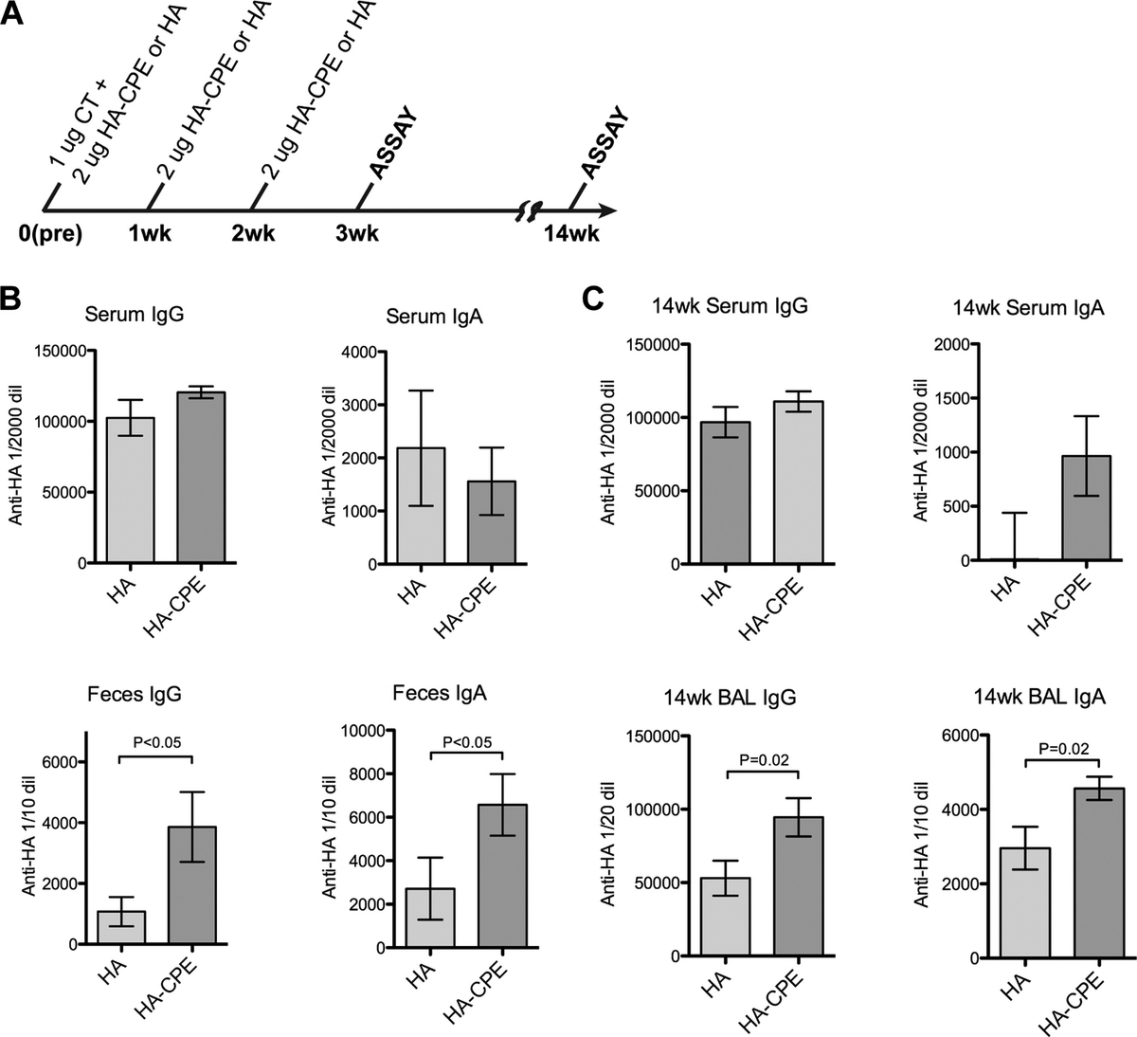
**Persistence of response to recombinant protein vaccine, three dose protocol**. Using an initial three week course of immunization followed by an eleven week rest period, persistent antibody responses were evident, along with enhanced IgA responses to the CPE targeted vaccine. A, Immunization protocol. B, Response 3 weeks after first dose. C, Response 14 weeks after first dose.

Similar results were found when a weekly three dose protocol (Figure 5A) was assessed for evidence for enhanced mucosal responses. In this case serum IgA was not significantly enhanced at the early time point (four weeks - one week after the last dose; Figure 5B) and the targeted vaccine showed the best mucosal response above background. The enhanced mucosal response showed persistence through the 14 week time point (Figure 5C). Fecal responses were not significantly higher in the group given the targeted vaccine at the 14 week time point (not shown), but as with the four dose protocol, lung bronchoalveolar lavage did show slightly enhanced responses in the group given the targeted vaccine (Figure 5C). Notably, as with the short term studies, the 14 week serum IgG responses were similarly strong in all groups, whether given non-targeted or targeted vaccine, or whether using a three dose or four dose protocol.

### Mucosal administration and Ig isotypes

**Figure 6:**
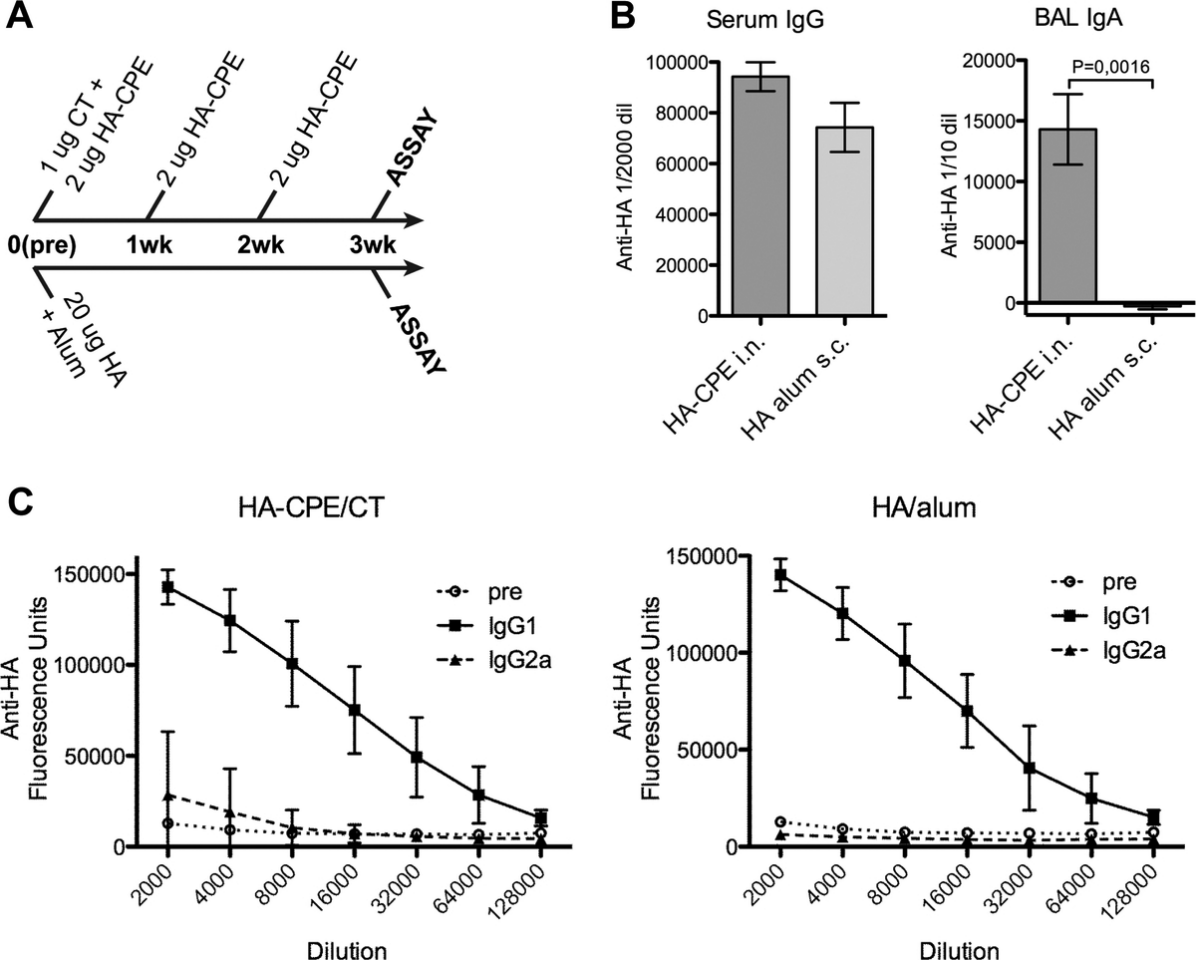
**Comparison of intranasal and subcutaneous immunization protocols**. Analysis of antibody responses to intranasal versus subcutaneous immunization shows similar Th2 dominance in the IgG isotype response. A, Immunization protocols showing the difference between the multiple dose intranasal administration and single dose subcutaneous alum administration. B, Differences in mucosal response to immunization showing the absence of mucosal responses in subcutaneously immunized mice despite similar systemic IgG responses. C, Titration of isotype specific ELISAs for anti-HA response showing the IgG1 dominance over IgG2a immune responses.

While targeted vaccine showed enhanced mucosal IgA responses, mucosal administration of non-targeted vaccine also induced some, albeit lower, mucosal IgA responses. By contrast, the serum IgG response to HA was generally equivalent whether targeted or not. Thus, intranasal administration and M cell targeting both contributed more to the induction of the enhanced mucosal IgA rather than to the systemic IgG response. To confirm that the administration route rather than the antigen itself was most important to IgA induction, we compared intranasal immunization with a conventional subcutaneous immunization given as a mixture with alum (Figure 6A).

Mice were given three weekly intranasal doses of 2 microgram HA-CPE with cholera holotoxin adjuvant in the first dose, or a single subcutaneous injection of 20 micrograms HA in alum. On the fourth week, both groups of mice showed similar serum IgG responses (Figure 6B). However, in mucosal tissues such as BAL, significant IgA anti-HA responses were present in mice given intranasal HA-CPE, while the HA/alum immunized mice had nearly undetectable IgA responses. Consistent with clinical responses to injected influenza vaccines, some mice did have low but detectable IgG responses in BAL (not shown).

The regulation of B cell isotype switching is determined by a combination of tissue specific factors (e.g., APRIL, TGF-beta) [26-29]. In the case of T cell dependent antibody responses, cytokines supporting IgA responses are predominantly associated with Th2 cells, also associated with a stronger IgG1 isotype response compared to an IgG2a isotype [30,31]. In confirmation of this Th2 dominance in the response to mucosal immunization, we found that the anti-HA titers in the serum showed an IgG1 dominance over IgG2a, estimated to be at least 10:1 (Figure 6C). This Th2 dominance may be a direct consequence of the mucosal immunization route [3], or the use of cholera toxin as the adjuvant [32,33]. Despite a similar Th2 dominance induced by alum adjuvant (Figure 6C), it clearly was not dependent on association mucosal IgA responses (Figure 6B). Moreover, the IgG titers were similar, though the antigen dose administered by the subcutaneous route (20 ug) was much higher than the total dose given by the intranasal route (6 ug). While it is also possible that the HA antigen is also predisposed toward Th2 responses, other forms of HA can induce Th1/IgG2a responses in mice [34].

## Discussion

The studies presented here extend our studies on M cell particle uptake showing that the Claudin 4 targeting CPE peptide can effectively mediate M cell uptake in both the NALT and intestinal Peyer's patches. In these studies, the enhanced uptake mediated specific enhancement of mucosal IgA responses, though the enhancement was not uniformly robust. Similar mouse M cell targeting of soluble proteins have been reported by others, both for inducing T and B cell immune responses including induction of secretory IgA [8-11,14] and for induction of mucosal tolerance [12,13]. A recent study successfully used the much larger toxin subunit C-CPE for mucosal vaccination [35], though in that study the authors expected the effect to depend on the C-CPE gaining entry past epithelial tight junctions. By contrast, our studies showed that our shorter CPE peptides do not disrupt epithelial tight junctions in vivo, and instead mediate uptake through M cells [20,21]. This approach using short CPE peptides is an advance over other strategies, as the targeting ligand has no risk of disrupting mucosal epithelium, and it can be used in humans (many other targeting approaches only apply to mouse M cells). While the short targeting peptide has only moderate affinity for the target receptor it does not appear to induce blocking antibodies, so it should be useful in multiple dosing strategies or for multiple vaccines. Our previous studies showed uptake of nanoparticles by M cells, but the present studies show that this can also be applied to the delivery of proteins in a physiological solution.

Interestingly, M cell targeting by CPE had little enhancing influence on serum IgG responses, suggesting that it may be more important in focusing antigen to mucosal lymphoid tissues for IgA induction rather than affecting the overall efficiency of antigen delivery. Since IgA isotype switching is thought to be mainly dependent on mucosal lymphoid tissues such as Peyer's patch, Isolated Lymphoid Follicles, and NALT, the utility of M cell targeting in mucosal vaccination may be specific to settings where secretory IgA is more desirable as the best form of protective immunity. In addition to local protection of mucosal epithelium from invasive microbes, targeted immunization for IgA responses can provide secondary benefits for mothers immunized against mucosal pathogens; IgA in the milk can help protect nursing infants [36]. Finally, mucosal vaccination in general also has clear value for its ease of administration, and efficiency in inducing persistent mucosal and systemic IgG responses on a per-dose basis.

## Conclusions

The present results extend our previous findings that a Claudin 4 targeting peptide can mediate enhance mucosal M cell uptake. Here, we found that fusion proteins incorporating both a vaccine antigen and a short Claudin 4-binding peptide can enhance mucosal IgA responses to intranasal administration. In addition, the intranasal route of vaccine administration appeared to be more efficient on a per-dose basis in inducing systemic IgG responses as compared to subcutaneous administration. Thus, mucosal vaccination strategies relying on targeting ligands such as the CPE peptide specific for known human M cell targets should have promise in clinical applications.

## Competing interests

The authors declare that they have no competing interests.

## Authors' contributions

DDL designed the experiments, performed the analysis, prepared the figures and wrote the manuscript. JL helped produce and characterize the recombinant proteins and performed peptide conjugations. HAE performed all of the ELISA assays and helped with experimental design and data analysis. All authors read and approved the final manuscript.
